# AntagomiR-103 and -107 Treatment Affects Cardiac Function and Metabolism

**DOI:** 10.1016/j.omtn.2018.12.010

**Published:** 2018-12-22

**Authors:** Monika Rech, Annika R. Kuhn, Joost Lumens, Paolo Carai, Rick van Leeuwen, Wouter Verhesen, Robin Verjans, Julie Lecomte, Yilin Liu, Joost J.F.P. Luiken, Ronny Mohren, Berta Cillero-Pastor, Stephane Heymans, Kèvin Knoops, Marc van Bilsen, Blanche Schroen

**Affiliations:** 1 CARIM School for Cardiovascular Diseases, Department of Cardiology, Maastricht University, 6229 ER Maastricht, the Netherlands; 2 CARIM School for Cardiovascular Diseases, Department of Physiology, Maastricht University, 6229 ER Maastricht, the Netherlands; 3 CARIM School for Cardiovascular Diseases, Department of Biomedical Engineering, Maastricht University, 6229 ER Maastricht, the Netherlands; 4 Department of Cardiovascular Sciences, Katholieke Universiteit Leuven, 3000 Leuven, Belgium; 5 CARIM School for Cardiovascular Diseases, Department of Molecular Genetics, Maastricht University, 6229 ER Maastricht, the Netherlands; 6 The Maastricht Multimodal Molecular Imaging Institute (M4I), Division of Imaging Mass Spectrometry, Maastricht University, 6229 ER Maastricht, the Netherlands; 7 The Maastricht Multimodal Molecular Imaging Institute (M4I), Microscopy CORE Lab, Maastricht University, 6229 ER Maastricht, the Netherlands; 8 Netherlands Heart Institute, 3511 EP Utrecht, the Netherlands

**Keywords:** Cardiomyocyte, cardiac, microRNA, miR-103, miR-107, antagomiR, mitochondria, metabolism, mass spectrometry, electron microscopy

## Abstract

MicroRNA-103/107 regulate systemic glucose metabolism and insulin sensitivity. For this reason, inhibitory strategies for these microRNAs are currently being tested in clinical trials. Given the high metabolic demands of the heart and the abundant cardiac expression of miR-103/107, we questioned whether antagomiR-mediated inhibition of miR-103/107 in C57BL/6J mice impacts on cardiac function. Notably, fractional shortening decreased after 6 weeks of antagomiR-103 and -107 treatment. This was paralleled by a prolonged systolic radial and circumferential time to peak and by a decreased global strain rate. Histology and electron microscopy showed reduced cardiomyocyte area and decreased mitochondrial volume and mitochondrial cristae density following antagomiR-103 and -107. In line, antagomiR-103 and -107 treatment decreased mitochondrial OXPHOS complexes’ protein levels compared to scrambled, as assessed by mass spectrometry-based label-free quantitative proteomics. MiR-103/107 inhibition in primary cardiomyocytes did not affect glycolysis rates, but it decreased mitochondrial reserve capacity, reduced mitochondrial membrane potential, and altered mitochondrial network morphology, as assessed by live-cell imaging. Our data indicate that antagomiR-103 and -107 decrease cardiac function, cardiomyocyte size, and mitochondrial oxidative capacity in the absence of pathological stimuli. These data raise concern about the possible cardiac implications of the systemic use of antagomiR-103 and -107 in the clinical setting, and careful cardiac phenotyping within ongoing trials is highly recommended.

## Introduction

The heart is the most metabolically demanding organ within the body due to its specialized function and limited capacity for energy substrate storage. In the healthy heart, approximately 70% of the energy demand is covered by the mitochondrial oxidation of fatty acids, while the remaining 30% stems from carbohydrates, ketones, lactate, and amino acids.^1^ The ATP generated in this way is mainly consumed by the myofibrillar actin-myosin ATPase for the contraction/relaxation of the cardiac muscle and by the Ca^2+^ ATPase for calcium reuptake in the sarcoplasmatic reticulum.^2^ Without tightly controlled energy supply and expenditure, cardiac function becomes severely compromised,^2^ and derangements of cardiac substrate and energy metabolism are considered to play a key role in the pathogenesis of heart failure. Indeed, therapies to prevent the progression of heart failure by improving the oxidative capacity of the heart are the subjects of recent investigations.^3^, ^4^

MicroRNAs (miRNAs) are a class of small non-coding RNA molecules that act as post-transcriptional modulators of gene expression; they are regulators of all major cell functions, including metabolism and growth;^5^ and they have been implicated in the development of cardiovascular disease.^6^ miRNAs have been shown to regulate cardiac hypertrophy and fibrosis.^7^, ^8^ Other miRNAs have been implicated in the regulation of metabolic pathways in general,^9^ or they have emerged as potential contributors to the metabolic substrate transition in cardiac diseases more specifically.^10^, ^11^, ^12^ Recently, the paralog miRNAs miR-103 and miR-107 were shown to regulate insulin resistance.^13^ Indeed, antagomiR-mediated silencing of miR-103/107 had marked effects on hepatic and adipose tissue metabolism, thereby significantly improving systemic glucose tolerance and insulin sensitivity in obese mice.^13^ These miRNAs are, therefore, considered promising therapeutic targets for the treatment of diabetes; a clinical phase I or IIA trial with antagomiR-103 and -107 to treat patients with type 2 diabetes and liver disease is currently ongoing (ClinicalTrials.gov: NCT02826525).

MiR-103/107 have been shown to be expressed at medium-to-high levels across many tissue types, including the heart.^14^ Importantly, the specific effects of antagomiR-103 and -107 in the heart have not been investigated yet. Considering the chief importance of balanced metabolism for the heart and the impact of miR-103/107 on systemic metabolism, it is essential to determine the role of miR-103/107 on cardiac function. Accordingly, in the present study, we investigated if antagomiR-mediated inhibition of miR-103/107 in mice had an impact on cardiac function and, if so, if this was related to alterations in cardiac metabolism. We found that antagomiR-103 and -107 treatment compromised mitochondrial function and led to cardiac structural and functional remodeling in the absence of any (patho)physiological trigger.

## Results

### AntagomiR-103 and -107 Treatment Impairs Cardiac Function in Healthy Adult Mice

**Figure 1:**
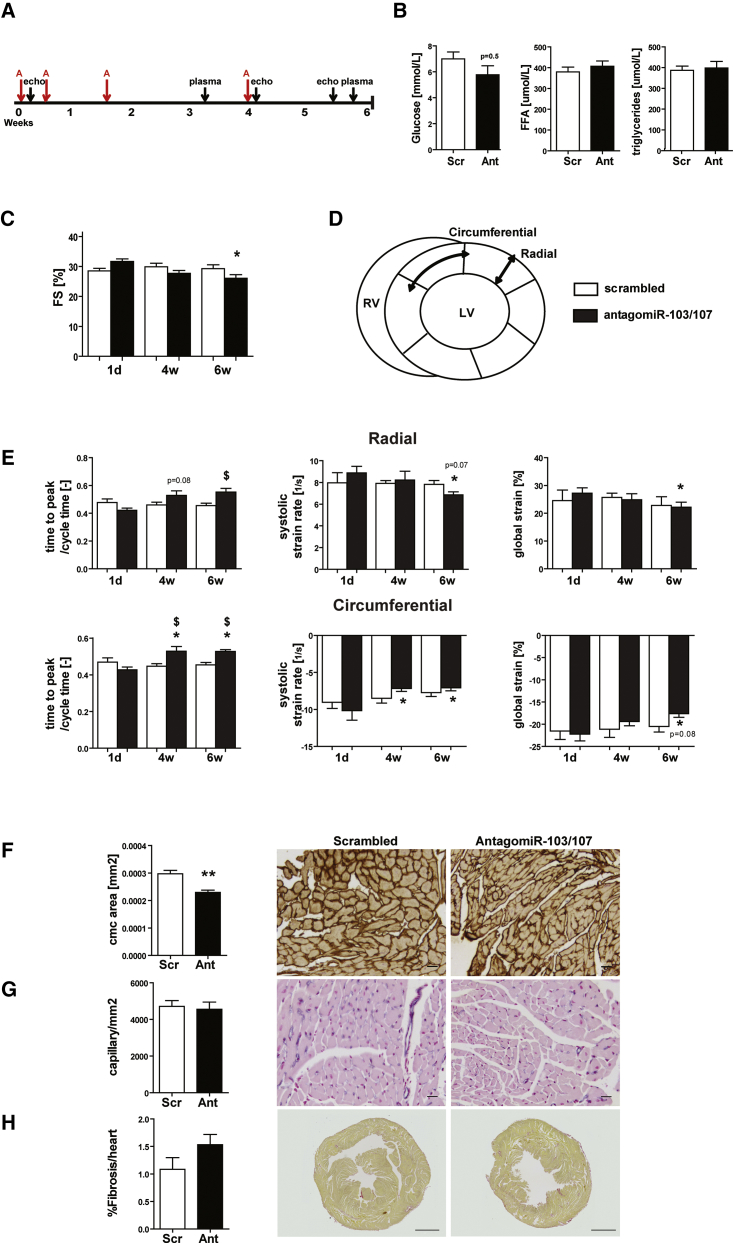
AntagomiR-103 and -107 Impair Cardiac Function and Strain (A) Timeline of the study. A, antagomiR-103 and -107. (B) Analysis of fasted plasma at week 3 of glucose, free fatty acids (FFAs), and triglycerides. (C) Echocardiographic fractional shortening (FS). (D) Schematic representation of the strain analysis, depicting the short-axis view of the left ventricle divided in the analyzed segments and indicating the radial and circumferential axes of motion. (E) Echocardiographic radial and circumferential strain and strain rate metrics. (F–H) Quantification and representative pictures of (F) laminin staining, indicating mean cardiomyocyte area (scale bar, 20 μm); (G) lectin staining, indicating capillary density (scale bar, 20 μm); and (H) picrosirius red staining, indicating fibrosis (scale bar, 1 mm). Scr, scrambled (40 mg/kg/day); Ant, antagomiR (20 mg/kg/day antagomiR-103 and 20 mg/kg/day antagomiR-107). Data are presented as mean ± SEM; n = 7 per group; *p < 0.05 versus scrambled; **p<0.01 versus scrambled; ^$^p<0.05 and p=x versus same treatment at day 1.

To study the effect of the inhibition of miR-103/107 on cardiac function and metabolism of unstressed hearts, antagomiR-103 and -107 was injected into adult mice (Figure 1A). After 6 weeks, the expression of both miRNAs was reduced by ∼70% in the heart and by >95% in the liver following antagomiR-103 and -107 treatment, compared to scrambled oligo-treated controls (Figure S1), demonstrating the effectiveness of the antagomiR-103 and -107 treatment. First, we explored the effect of the antagomiR-103 and -107 on systemic metabolism, and we monitored circulating free fatty acid, triglyceride, and glucose levels over the course of 6 weeks. In line with a previous study,^13^ fasting glucose levels tended to decrease in the antagomiR-103 and -107 group compared to control after 3 weeks (Figure 1B). At the same time, the levels of free fatty acids and triglycerides were unchanged between groups (Figure 1B). Hematological analysis at sacrifice showed a significant decrease in total white blood cell counts and lymphocyte counts in the antagomiR-103 and -107 group as compared to scrambled controls (Table S1). Differences in neutrophil and monocyte counts were not observed (Table S1). Morphometric parameters did not show differences between experimental groups (Table 1). These data indicate that antagomiR-103 and -107 only had mild effects on systemic metabolism in healthy, non-obese mice.

**Table 1:** Morphometric Parameters at Sacrifice

Morphometrics	Scrambled		AntagomiR-103 and -107	
	Mean	±SD	Mean	±SD
Heart weight/tibia length (mg/mm)	6.00	0.157	6.05	0.228
Liver weight/tibia length (mg/mm)	65.13	1.533	64.00	1.460
Kidney weight/tibia length (mg/mm)	8.90	0.188	8.87	0.351
Lung weight/tibia length (mg/mm)	8.88	0.226	9.46	0.258
Spleen weight/tibia length (mg/mm)	5.17	0.195	5.77	0.202
Body weight (week 0)	22.00	0.415	22.42	0.513
Body weight (week 6)	26.00	0.388	25.98	0.348

n = 7 per group; statistical differences were not observed.

To monitor cardiac effects of the inhibition of the two miRNAs, left ventricle (LV) systolic function was evaluated by echocardiographic analysis at 1 day, 4 weeks, and 6 weeks after the start of antagomiR-103 and -107 treatment. At day 1 and week 4, cardiac fractional shortening (FS) was comparable between groups (Figure 1C). Remarkably, at week 6, the antagomiR-103 and -107 group presented a modest but significant reduction in FS compared to scrambled control. To gain more detailed information on the dynamics of myocardial contraction in the LV, we performed speckle-tracking strain analysis, and we assessed global LV circumferential and radial strains as well as time to peak (TTP) systolic strain and peak systolic strain rates in both dimensions (Figures 1D and 1E). In line with the decreased FS at 6 weeks, antagomiR-103 and -107 treatment led to a prolonged TTP in both radial and circumferential dimensions after 4 and 6 weeks compared to day 1 and for the circumferential dimension also compared to scrambled control (Figure 1E). However, the scrambled control group did not show a difference in TTP over the time of the study. Consistent with the decreased LV systolic function observed in terms of FS, ejection fraction (EF), and TTP (Figure 1E; Table 2), 6 weeks of antagomiR-103 and -107 treatment led to decreased global strain in both radial and circumferential planes compared to day 1 (Figure 1E). AntagomiR-103 and -107 treatment also led to reduced peak systolic radial strain rate after 6 weeks and peak systolic circumferential strain rate after 4 and 6 weeks (Figure 1E). Remarkably, the inhibition of miR-103/107 led to decreased cardiomyocyte size without modifying the capillary density and cardiac fibrosis, as assessed by histological analysis (Figures 1F–1H).

**Table 2:** Echocardiographic Parameters

Parameter	Day 1					Week 4				Week 6				
	Scrambled			AntagomiR-103 and -107		Scrambled		AntagomiR-103 and -107		Scrambled			AntagomiR-103 and -107	
	Mean	± SD	Mean		± SD	Mean	± SD	Mean	± SD	Mean	± SD	Mean		± SD
Average wall thickness (mm)	0.77	0.09	0.86*		0.06	0.83	0.06	0.77*	0.10	0.83	0.13	0.82		0.07
Heart rate (bpm)	546.71	39.00	583.00		42.00	548.29	30.00	570.00	32.00	532.00	36.00	576.00*		29.00
LV volume diastolic (μL)	28.34	3.98	25.40		7.12	34.60	7.62	40.69	7.33	35.58	8.59	39.13		9.50
LV volume systolic (μL)	63.78	7.68	63.52		14.48	80.19	10.25	88.08	13.45	80.68	12.82	80.67		18.78
Stroke volume (%)	35.44	4.66	38.12		7.78	45.59	4.25	47.39	7.50	45.10	5.98	41.54		10.52
Ejection fraction (%)	55.60	3.35	60.31		3.15	57.22	4.92	53.84	3.89	56.25	5.33	51.43*		4.62
Fractional shortening (%)	28.50	2.23	31.66		2.13	29.85	3.23	27.67	2.47	29.23	3.49	26.06*		2.91
Circ. TTP/cycle time (−)	0.47	0.06	0.44		0.07	0.45	0.04	0.52*^$^	0.05	0.45	0.03	0.52*^$^		0.05
Radial TTP/cycle time (−)	0.48	0.06	0.43		0.07	0.46	0.04	0.53	0.05	0.45	0.03	0.56$		0.05
Global circ. strain (%)	−21.53	5.05	−22.22		3.74	−21.16	4.75	−19.38	2.35	−20.52	3.24	−17.62*		2.04
Global radial strain (%)	24.54	10.08	26.91		3.95	25.68	4.19	26.67	6.24	22.84	8.22	21.76*		3.69
Circ. strain rate diastolic (1/s)	10.18	1.54	9.50		1.34	10.38	1.96	10.79	1.35	8.81	1.49	9.53		0.64
Circ. strain rate systolic (1/s)	−9.04	2.19	−9.87		2.69	−8.50	1.66	−7.24*	0.77	−7.73	1.39	−7.14*		0.83
Radial strain rate diastolic (1/s)	9.27	2.35	9.13		2.35	9.95	1.83	12.03*^$^	1.35	7.74	2.10	10.11		1.88
Radial strain rate systolic (1/s)	7.98	2.45	8.89		1.50	7.92	0.67	8.23	1.99	7.82	0.99	6.86*		0.71

n = 7 per group; *p < 0.05 versus scrambled, ^$^p < 0.05 versus same treatment at day 1. circ., circumferential.

Taken together, chronic treatment with antagomiR-103 and -107 led to a significant decrease of LV systolic function paralleled by a prolonged TTP LV contraction and a decreased systolic strain rate, in the absence of any (patho)physiological trigger.

### AntagomiR-103 and -107 Do Not Affect the Inotropic State

**Figure 2:**
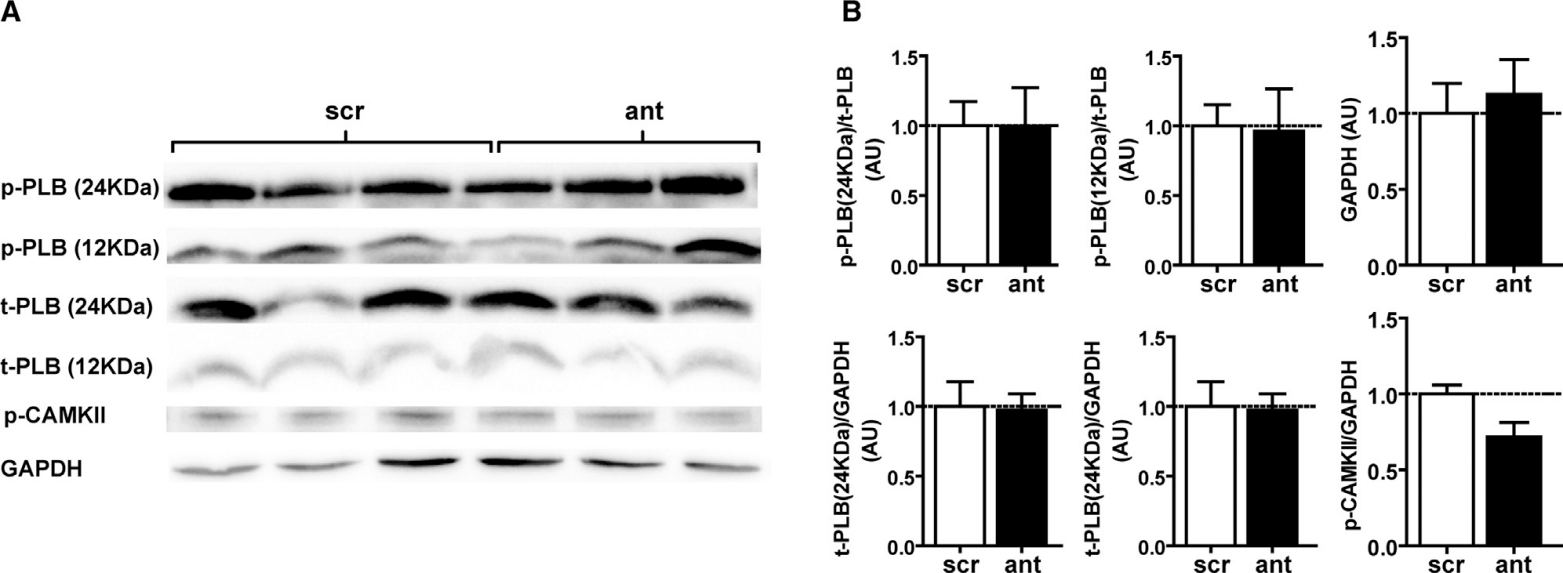
AntagomiR-103 and -107 Do Not Affect Phosphorylation of Proteins Involved in Cardiac Ca^2+^ Homeostasis (A) Representative western blot of the lysates of hearts treated with antagomiR-103 and -107 or scrambled. (B) Quantification of western blot. Scr, scrambled; ant, antagomiR. Data are presented as mean ± SEM; n = 3 per group.

The echocardiographic strain analysis, which showed prolonged TTP and decreased systolic strain rate upon miR-103/107 inhibition, may point toward a negative inotropic effect of antagomiR-103 and -107. Negative inotropic agents can decrease myocardial force and contractility by slowing cardiomyocyte Ca^2+^ flux or by reducing Ca^2+^ sensitivity of sarcomeric proteins via phosphorylation or dephosphorylation. However, the phosphorylation status of phospholamban (PLB) and cardiac troponin I (p-TnI), key regulators of sarcoplasmic reticulum Ca^2+^ sequestration and myofilament Ca^2+^ sensitivity that become phosphorylated upon inotropic stimulation, was not significantly changed upon antagomiR-103 and -107 treatment (Figures 2A and 2B). In addition, phosphorylation of Ca^2+^/calmodulin-dependent protein kinase II (p-CaMKII), essential for Ca^2+^ homeostasis and for the regulation of the contraction-relaxation cycle, did not differ between antagomiR-103 and -107-treated and control mice (Figures 2A and 2B). These results indicate that the observed effect of antagomiR-103/107 treatment on myocardial strain is not likely to be due to alterations in inotropic state.

### AntagomiR-103 and -107 Modifies the Cardiac Proteome

To gain insight into underlying processes that could drive cardiac dysfunction and remodeling upon the inhibition of miR-103/107, we performed a miR-103/107 target analysis. According to the online database TargetScan,^15^ miR-103/107 have 838 predicted targets, which can be grouped according to their biological function using the PANTHER gene annotation database^16^ (Figure S2). Interestingly, ∼29% of anticipated miR-103/107 targets are associated with metabolic processes, including carbohydrate as well as lipid metabolism.

**Figure 3:**
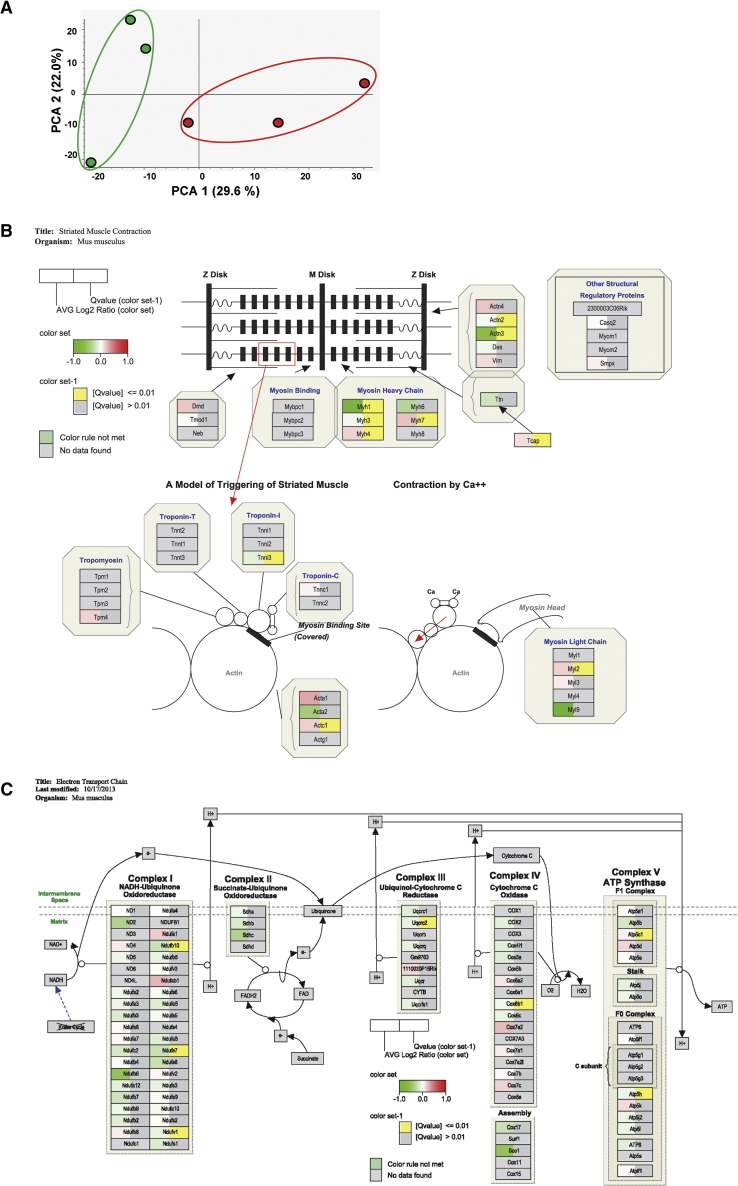
AntagomiR-103 and -107 Modify the Cardiac Proteome (A) Unsupervised mass spectrometry-protein abundancies clustering resulting from the multivariate analysis (principal-component analysis [PCA]) by the software Proteome Discoverer 2.2 (Thermo Scientific). Green, scrambled (n = 3); red, antagomiR-103 and -107 (n = 3). (B and C) PathVisio visualization of (B) striated muscle contraction and (C) ETC protein levels quantified by mass spectrometry of heart lysates. Green coloring indicates less expression of protein in antagomiR-103 and -107-treated mice, red coloring indicates an increased expression, and yellow coloring indicates a significant q-value (q < 0.01).

To further probe the mechanism underlying the antagomiR-103 and -107-induced alterations in cardiac function, we performed mass spectrometry (MS)-based label-free quantitative proteomics on LV tissue from scrambled and antagomiR-103 and -107-treated mice. Unsupervised clustering analysis of the MS data clearly separated the scrambled control and the antagomiR-103 and -107 group (Figure 3A), underscoring the effect of antagomiR-103 and -107 on cardiac phenotype. Anticipated targets of miR-103/107, including mitochondrial proteins, were differentially expressed upon antagomiR-103 and -107 treatment (Table S2). The generated data were integrated into the pathway analysis application PathVisio for the visualization of differentially regulated pathways related to sarcomere function and metabolism. Multiple proteins involved in striated muscle contraction as well as the mitochondrial electron transport chain (ETC) were found to be differentially expressed following antagomiR-103 and -107 treatment (Figures 3B and 3C; Table S2). Indeed, antagomiR-103 and -107 affected protein levels within the ETC complexes I, III, IV, and V as compared to scrambled control (Figure 3C).

### AntagomiR-103 and -107 Decrease Cardiomyocyte Mitochondrial Volume Density

**Figure 4:**
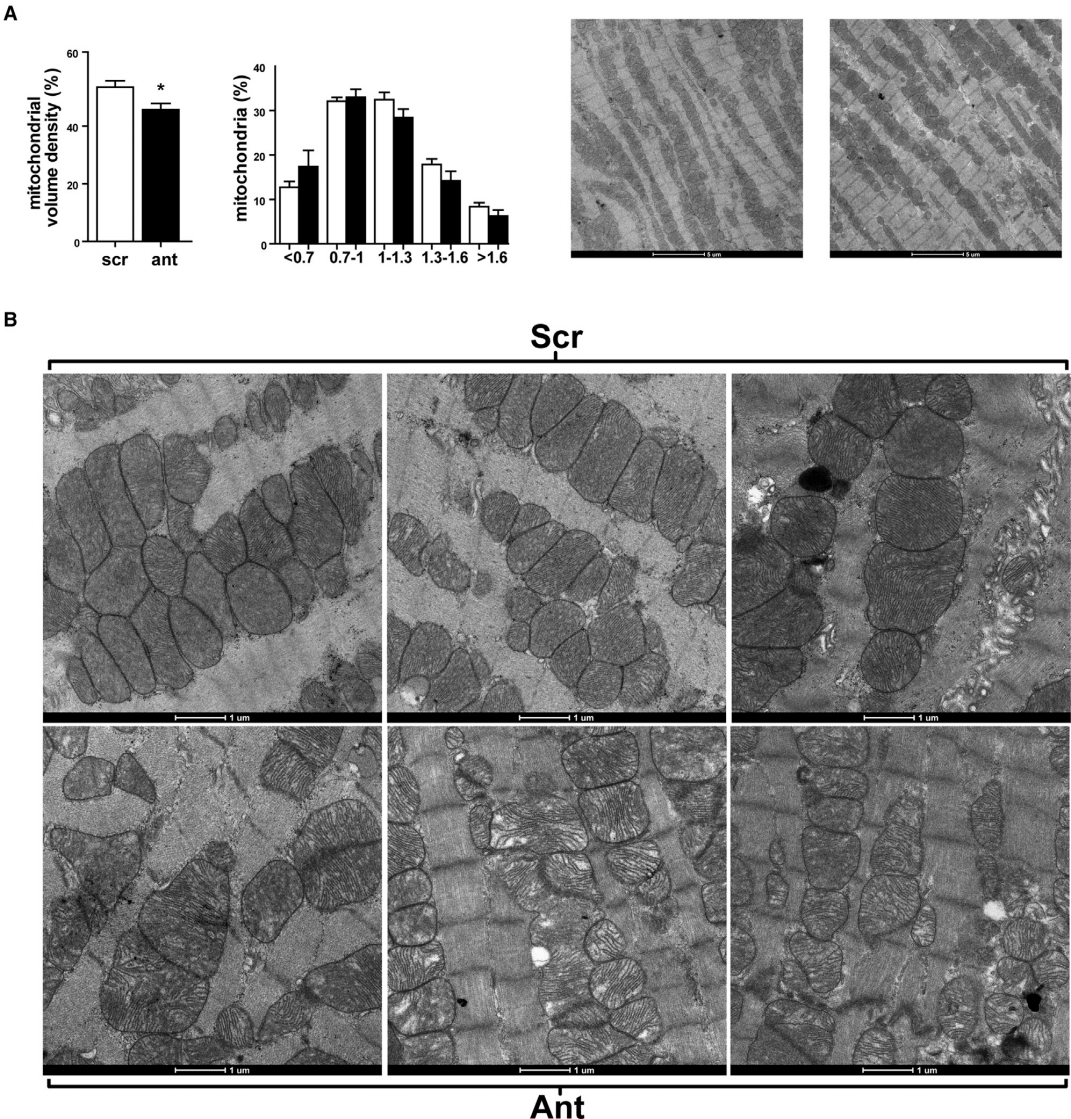
AntagomiR-103 and -107 Decrease Cardiac Mitochondrial Volume Density (A) Mitochondrial volume density and size range quantification. TEM images show cardiac tissue of antagomiR-103 and -107- or scrambled-treated hearts at 1,900× magnification (n = 5 per group). Data are presented as mean ± SEM; *p < 0.05 versus scrambled. (B) TEM images visualizing mitochondrial morphology of antagomiR-103 and -107- or scrambled-treated hearts at 6,800× magnification. Scr, scrambled; Ant, antagomiR.

In view of the reported role of miR-103/107 in regulating metabolism,^17^ the prominence of predicted targets related to metabolism (Figure S2), and our MS data on the ETC, we decided to focus on the effects of miR-103/107 inhibition on cardiac energy metabolism, as a possible explanation for the decline in cardiac function. As a first general characterization of cardiac energy status, we investigated the number and size distribution of the mitochondria within cardiomyocytes by means of transmission electron microscopy (Figure 4A). Notably, mitochondrial volume density was significantly lower in the antagomiR-103 and -107-treated group as compared to scrambled control. The number of mitochondria was not changed significantly, but there was a general trend toward smaller-sized mitochondria in the antagomiR-103 and -107-treated group compared to scrambled control (p = 0.1; Figure 4A). In the high-resolution images, mitochondrial morphology was visibly altered in the antagomiR-103 and -107-treated group, as revealed by the mitochondrial cristae appearance (Figure 4B). Together, the negative effects of antagomiR-103 and -107 on mitochondrial volume density and on ETC protein expression may point to a reduction in cardiac mitochondrial capacity following antagomiR-103 and -107 treatment.

### miR-103/107 Inhibition in Cardiomyocytes Reduces Mitochondrial Respiration

**Figure 5:**
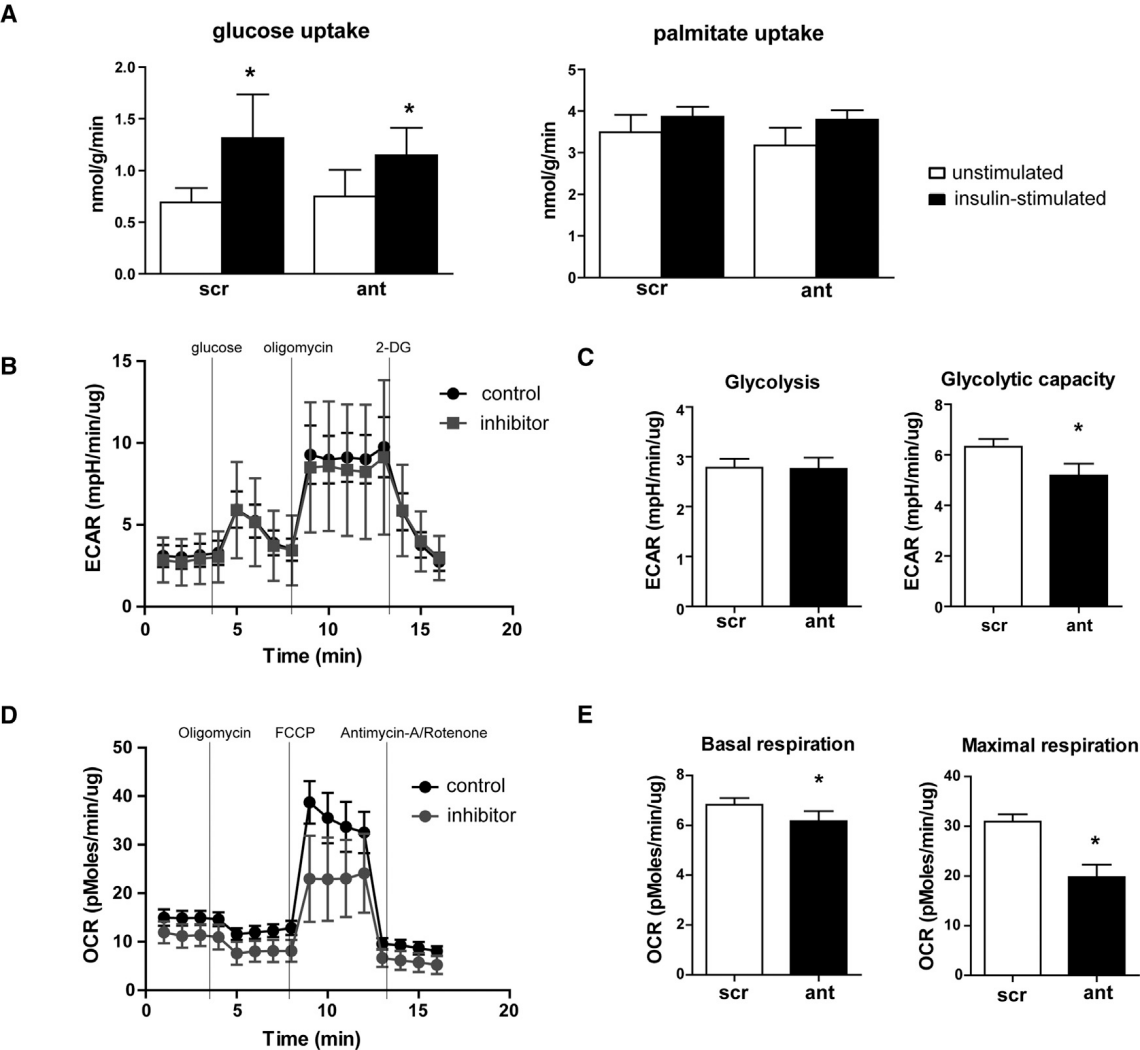
miR-103/107 Inhibition in Cardiomyocytes Decreases Mitochondrial Respiration (A) Bar graphs of insulin-stimulated glucose and palmitate uptake of HL-1 cells transfected with scrambled control or inhibitors for miR-103/107. (B) XF trace of the extracellular acidification rate (ECAR) of nRCM transfected with scrambled control or inhibitors for miR-103/107. (C) Glycolysis and glycolytic capacity quantification from (B). (D) XF trace of the oxygen consumption rate (OCR) of nRCM transfected with scrambled control or inhibitors for miR-103/107. (E) Quantification of mitochondrial basal and maximal respiration from (D). 2-DG, 2-deoxyglucose; FCCP, Carbonyl cyanide p-trifluoromethoxyphenylhydrazone; scr, scrambled; ant, inhibitors. Data are presented as mean ± SEM of at least 4 replicates; *p < 0.05 versus scr.

Given the effects of *in vivo* antagomiR-103 and -107 treatment on cardiac mitochondrial indices, we next investigated the effects of miR-103/107 inhibition on substrate uptake and energy metabolism in cardiomyocytes *in vitro*. First, we measured the degree of insulin-stimulated glucose and palmitate uptake in HL-1 cardiomyocytes. In line with our previous results,^18^ insulin stimulation induced the uptake of glucose and tended to induce palmitate uptake in HL-1 cardiomyocytes (Figure 5A). Importantly, miR-103/107 inhibition did not significantly change the level of glucose or palmitate uptake as compared to control, neither under basal nor under insulin-stimulated conditions (Figure 5A). These data indicate that the inhibition of miR-103/107 in cardiomyocytes does not alter the substrate uptake rate in cardiomyocytes.

To assess the effects of miR-103/107 on energy expenditure, substrate utilization, and mitochondrial respiration, neonatal rat cardiac myocytes (nRCMs) were transfected with inhibitors for miR-103/107 or negative control, and metabolic assessment was conducted by metabolic flux analysis (Seahorse). miR-103/107 inhibition did not affect the extracellular acidification rate (ECAR), an indirect measure for glycolysis, but it decreased glycolytic capacity as compared to control (Figures 5B and 5C). miR-103/107 inhibition significantly decreased the oxygen consumption rate (OCR), a measurement of mitochondrial respiration, at baseline and even more so after maximal stimulation by Carbonyl cyanide p-trifluoromethoxyphenylhydrazone (FCCP) (Figures 5D and 5E), as reflected by lower basal and maximal respiration (Figure 5E). These data suggest that the inhibition of miR-103/107 decreases mitochondrial respiration in cardiomyocytes.

**Figure 6:**
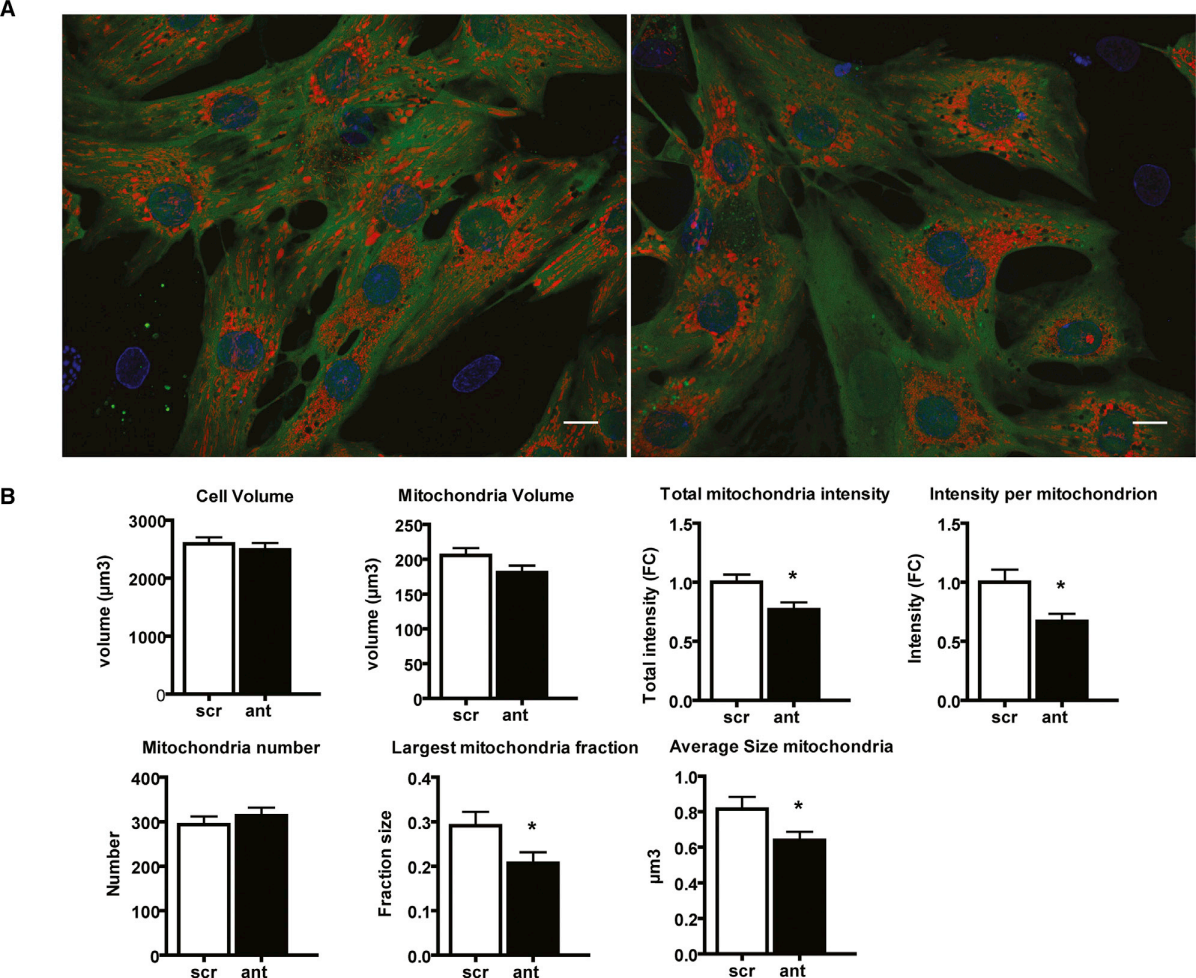
miR-103/107 Inhibition in Cardiomyocytes Affects Mitochondrial Morphology (A) Maximum-intensity projections of nRCMs stained with calcein (green), Hoechst (blue), and TMRM (red) to visualize cytosol, nuclei, and mitochondria, respectively. Scale bar, 10 μm. (B) Mitochondrial network analyses including cell and mitochondria volume, mitochondrial intensities (reflecting mitochondrial membrane potential) as measured by TMRM fluorescence, and quantification of mitochondrial number and size. Scr, scrambled; ant, inhibitors. Data are presented as mean ± SEM of 40 individually analyzed cells per condition; *p < 0.05 versus scr.

Additionally, to investigate the effects of miR-103/107 inhibition on mitochondrial function and morphology, nRCMs were transfected with inhibitors for miR-103/107 or negative control and stained with Tetramethylrhodamine methyl ester (TMRM) to monitor mitochondrial membrane potential and mitochondrial network structure. We quantified TMRM signal intensity, and we assessed mitochondrial morphology three-dimensionally in live nRCMs with spinning-disc microscopy. miR-103/107 inhibition did not significantly affect cell volume, mitochondrial volume, or number of mitochondria (Figures 6A and 6B). Reduced total mitochondria intensity as well as intensity per mitochondrion were indicative of changes in mitochondrial membrane potential due to the inhibition of miR-103/107 (Figure 6B). In addition, mitochondrial networks were more fragmented after miR-103/107 inhibition, as reflected by significantly smaller largest mitochondrial fractions. Finally, individual mitochondria were smaller in nRCM following the inhibition of miR-103/107 (Figure 6B).

These results are in line with our *in vivo* data on decreased sarcomeric mitochondrial volume density as well as changes in mitochondrial morphology, ETC protein levels, and the mild reduction in cardiac function.

Taken together, our data show that chronic inhibition of miR-103/107 in healthy mice compromises mitochondrial function and leads to cardiac structural and functional remodeling.

## Discussion

The protective function of antagomiR-103 and -107 on systemic glucose metabolism and insulin sensitivity has been recently established for metabolic disease.^13^ In this study, we applied antagomiR-103 and -107 and disclosed their effect on cardiac function and cardiomyocyte metabolism. We showed that systemic delivery of antagomiR-103 and -107 in healthy mice reduced cardiac LV function, as indicated by decreased FS and strain rate in the absence of pathological stimuli. In addition, antagomiR-103 and -107-treated mice presented with a diminished cardiac mitochondrial volume density as well as decreased protein levels of the striated muscle contraction and the ETC complexes. The investigation of cardiomyocyte metabolism upon miR-103/107 inhibition showed a reduced mitochondrial respiratory capacity. Our results suggest that miR-103/107 inhibition affects cardiac systolic function via the modulation of mitochondrial respiration, without affecting the overall ATP availability for the cardiomyocytes. Which one(s) of the predicted targets of miR-103/107 is responsible for mediating the observed effect on cardiac function remains to be established.

miR-103 and miR-107 are conserved in all known vertebrate species and are paralogs that differ only at a single nucleotide, and, hence, they are thought to have overlapping targets.^17^ miR-103 and miR-107 are located within introns of genes coding for pantothenate kinases (PANKs). PANKs are enzymes that regulate cellular coenzyme A levels, affecting multiple metabolic reactions, including the synthesis of fatty acids, amino acids, cholesterol, pyruvate, glucose, and tricarboxylic acid (TCA) cycle intermediates.^17^ Based on the fact that miRNA expression is often correlated with their host gene expression, it has been proposed that miR-103/107 also play a significant role in PANK-associated metabolic reactions.^17^ Moreover, bioinformatics target prediction indicated that miR-103/107 target exceptionally more metabolic enzymes than usually seen from miRNAs. These predictions include fatty acid synthase, carnitine palmitoyl transferase I, and pyruvate dehydrogenase.^17^ Accordingly, using TargetScan^15^ and PANTHER databases,^16^ we found that ∼29% of anticipated miR-103/107 targets are associated with metabolic processes, including carbohydrate as well as lipid metabolism. In the current study, we were not able to detect significant changes in cardiac metabolic gene expression at the mRNA level following antagomiR-103 and -107 treatment. However, in line with a previous study,^13^ these regulatory effects could be detected in liver tissue (data not shown), the main tissue targeted by antagomiR nucleotides. Since miR-103/107 were incompletely inhibited in the heart (about 70%), it is conceivable that some residual regulation by miR-103/107 still took place. However, this remains to be further investigated.

Increased expression of miR-103/107 in liver has been associated with insulin resistance in patients with alcoholic liver disease, nonalcoholic fatty liver disease, and nonalcoholic steatohepatitis, conditions often associated with diabetes.^19^ Moreover, previous studies of miRNA microarray analysis, aimed at selecting the most deregulated miRNAs in obesity and insulin resistance, found miR-103/107 to be among the most upregulated in the livers of two types of obese mice, ob/ob and diet-induced obese mice,^13^ and the expression of these miRNAs was also reportedly increased in diabetic Goto-Kakizaki rats.^20^ These data indicated an association of miR-103/107 with insulin resistance, which led to the idea of using antagomiRs against miR-103/107 as an anti-diabetic drug.^13^ Liver-specific overexpression of miR-103/107 in mice induced hyperglycemia and hyperinsulinemia, and also it impaired glucose tolerance,^13^ suggesting that increased miR-103/107 levels during liver disease associated with diabetes may contribute to disease progression. Conversely, antagomiR-103 and -107 treatment in obese mice improved glucose tolerance and insulin sensitivity in liver and adipose tissue, and it rescued β-oxidation pathway genes like *Cpt1*.^13^ Currently, a clinical phase I or IIa trial is ongoing, which applies antagomiR-103 and -107 in patients with steatohepatitis and type 2 diabetes. In line, in our study antagomiR-103 and -107 treatment led to a trend to decreased plasma glucose in healthy animals.

To study the myocardial deformation pattern, we performed speckle-tracking echocardiography analysis. Speckle-tracking echocardiography is widely used in the clinical setting for the assessment of global and regional systolic function in patients with heart failure and apparently normal EF.^21^ While EF and FS are parameters that describe the myocardial pump function, strain and strain rate quantify the contractility pattern of the myocardium.^22^, ^23^ In the present study, while the systolic strain rate decreased at weeks 4 and 6 in the antagomiR**-**103 and -107-treated group, the diastolic strain rate was increased at week 4 in the radial plane. This suggests a possible partial compensation for the prolonged systolic phase, as indicated by TTP. These measurements fit with a normal physiological range of LV function in which the heart is able to compensate for a prolonged systolic phase with a higher strain rate of relaxation. These findings are particularly important since antagomiR-103 and -107 are currently being investigated for clinical application in a phase I or IIa clinical trial. Our results may be indicative of an effect on cardiac function within the patient population of the ongoing clinical trial, which had originally been enrolled for the presence of liver metabolic disease. In this context, it is noteworthy that the current findings are derived from studies with healthy, unstressed animals. It is well feasible that the observed depression of cardiac function may be even more pronounced in the challenged heart.

Among the physiological modifiers of cardiac contractile force and speed of contraction, we investigated the effect of antagomiR-103 and -107 on the cardiac inotropic state, by measuring the phosphorylation status of proteins involved in Ca^2+^ homeostasis. Phospholamban (PLB) modulates sarcoplasmic reticiulum Ca^2+^ uptake by inhibiting sarco/endoplasmic reticulum Ca^2+^-ATPase (SERCA),^24^ and it becomes phosphorylated upon cyclic AMP (cAMP)-protein kinase A (PKA) stimulation. We did not detect changes in PLB phosphorylation or total PLB (t-PLB) in antagomiR-103 and -107-treated mice as compared to controls. Moreover, CaMKII signaling is central in cardiac contractility and calcium handling.^25^ Activated and phosphorylated CaMKII, associated with the sarcoplasmic reticulum, regulates both calcium uptake and release functions with each cycle of contraction and relaxation.^25^ We analyzed cardiac protein levels of total TnI (t-TnI) and p-TnI, and we did not find a difference between antagomiR-103 and -107 treatment and control. Although there was no definitive proof, the absence of changes in phosphorylation of these proteins suggests that the observed decline in cardiac function is not secondary to alterations in inotropic state.

Interestingly, antagomiR-103 and -107 treatment led to changes in contractile protein expression, as assessed by MS, and it was associated with a reduction in cardiomyocyte size. Together, this points to an active cardiac remodeling process induced by the antagomiR-103 and -107 treatment. Despite smaller cardiomyocytes following antagomiR-103 and -107 treatment, total heart weight and cardiac wall dimensions were not affected, which might imply an increased volume of the interstitial space and the presence of edema. It remains to be established if these observations are a reflection of atrophic or apoptotic processes^26^ and, importantly, if this contributes to the decline in cardiac function.

The impairment of bioenergetics is considered key in the development of heart failure (HF).^27^ The ATP that the myocardium has to synthesize and transfer to sustain the excitation-contraction coupling must continuously support an optimal myocardial performance in both systolic and diastolic phases.^27^ ATP availability is crucial for Ca^2+^ homeostasis during the excitation-contraction cycle,^28^ and most of the ATP derives from mitochondrial oxidative phosphorylation. In light of the current knowledge and of our *in vivo* data on the decreased cardiac levels of multiple proteins of the mitochondrial ETC, one possible explanation for the observed contractile dysfunction in this study is that, in antagomiR-103 and -107-treated hearts, mitochondrial function, and thus ATP synthesis, gets compromised. The effects of miR-103/107 inhibition on mitochondrial membrane potential, an indicator of mitochondrial health, as shown by the live-cell imaging of the uptake of TMRM and the increased mitochondrial network fragmentation, are in support of this reasoning. Since calcium is an important signaling molecule involved in the ATP-consuming cardiac muscle contraction and relaxation,^22^ future investigations should consider contractility readouts in cardiomyocytes of mice treated with antagomiR-103 and -107.

A validated target of miR-103/107 is caveolin-1 (Cav-1),^13^ the principal structural protein of caveolae, which are membrane domains that have been implicated in signal transduction.^29^, ^30^ Trajkovski and colleagues^13^ have shown that Cav-1 upregulation is paralleled by a concomitant stabilization of the insulin receptor. Moreover, Meshulam and colleagues^31^ have shown that the expression of Cav-1 facilitates the uptake of fatty acids. Indeed, caveolae have been suggested to participate in lipid transport, and they may serve as organizing centers for a variety of receptors and signal-transducing molecules.^32^ In light of this function of Cav-1, we have studied the substrate uptake of HL-1 cardiomyocytes upon the inhibition of miR-103/107, and we found that the substrate uptake was not affected upon the inhibition of miR-103/107 at baseline.

### Conclusions

The current findings highlight the role of miR-103/107 in cardiac function. We show that systemic inhibition of miR-103/107 by means of antagomiRs decreases cardiac systolic function, as reflected by the reduced FS and systolic strain rate, in the absence of pathological stimuli. In addition, antagomiR-103 and -107 led to decreased cardiac mitochondrial volume density, altered mitochondrial morphology, and reduced mitochondrial OXPHOS protein content and membrane potential. The present findings raise concern on the possible cardiac implications for the use of antagomiR-103 and -107 as an anti-diabetic drug, and careful cardiac phenotyping within ongoing clinical trials is highly recommended.

## Materials and Methods

### Animals

All animal experiments were approved by the Institutional Animal Care and User Committee of Maastricht and Leuven Universities, and they were performed adhering to the Dutch and Belgian law. C57BL/6J male mice were included in the experiment at the starting age of 7 weeks (Figure 1A). C57BL/6J mice (7/group) were injected with either scrambled RNA (40 mg/kg/day) or antagomiR-103 and -107 (40 mg/kg/day) at experimental days 0, 1, 8, and 29. AntagomiRs were RNA oligos complementary to miR-103 and miR-107, 2′-0-methyl modified, harboring a 3′-cholesterol type 1 modification and 5–7 phosphorothioate internucleotide linkages. Sequences were as follows: antagomir miR-107-3p, 5′-U*G*AUAGCCCUGUACAAUGCU*G*C*U*Chol*T-3′; antagomir miR-103a1-3p, 5′-U*C*AUAGCCCUGUACAAUGCU*G*C*U*Chol*T-3′; and antagomir scrambled, 5′-C*A*GCUGAAGUAAAUACCGAC*C*A*G*Chol*T-3′ (Fidelity Systems, Gaithersburg, MD).

Systolic blood pressure was monitored at weeks 3 and 6 by means of tail cuff (CODA, Kent Scientific, Torrington, CT). Glycemia was assessed at day 21 in plasma after a 6-h fast. Cardiac function was assessed by echocardiography at days 0, 28, and 41 under isoflurane anesthesia (mean 1% in oxygen) with the Vevo model 2100 from FUJIFILM (VisualSonics, Amsterdam, the Netherlands), with probe MS400 18–38 MHz. For the duration of the study, mice were housed in groups of four to five in ventilated cages in a temperature-controlled room with a 12-h light-dark cycle and *ad libitum* access to food and water. At day 42, animals were euthanized; blood and organs were processed for electron microscopy and histological and molecular analyses.

### Echocardiographic Analysis

Echocardiographic M-mode and B-mode images were acquired in mid-ventricular views at the height of the papillary muscles. Heart rate was obtained from the short axis B-mode. LV dimensions were measured in systole and diastole. Cardiac strain measurements were obtained from parasternal short-axis B-mode images using the VevoStrain software (Visualsonics). Images with clear visibility of endocardial and epicardial borders throughout the cardiac cycle and with minimal image artifacts were selected for strain analysis. Three to four consecutive cardiac cycles were selected, then endocardial and epicardial borders were traced semi-automatically and manually adjusted for tracking during the cardiac cycle. Using speckle-tracking-based strain analysis, circumferential and radial strain and strain rate time curves were quantified in six adjacent segments. Frame rate was 333/s for all 2D B-mode acquisitions. Several LV systolic function metrics were obtained from the circumferential and radial strain and strain rate time curves. TTP shortening was defined as the time from onset QRS to peak systolic shortening, averaged over all segments. Furthermore, global circumferential and radial strains (GCS and GRS, respectively) were obtained by averaging all segmental peak systolic strain values. Circumferential and radial peak systolic strain rates were defined as the maximum shortening and thickening velocity between onset QRS and time of peak systolic strain, respectively, averaged over all segments.

### RNA and RT-PCR

Cardiac tissue was lysed with stainless steel beads (QIAGEN, Venlo, the Netherlands) in lysis buffer with the TissueLyser LT (QIAGEN, Venlo, the Netherlands). RNA was isolated from the lysate with mirVANA kit (Thermo Fisher Scientific, Waltham, MA), according to the manufacturer’s instructions. DNase treatment was performed with DNAfree kit (Thermo Fisher Scientific, Waltham, MA), according to the manufacturer’s guidelines. Reverse transcription was performed with miScript kit (QIAGEN, Venlo, the Netherlands), using equal RNA input according to the manufacturer’s instructions. RT-PCR was performed with iQ SYBR Green Supermix (Bio-Rad, Hercules, CA) and iCycler iQ (Bio-Rad, Hercules, CA), according to the manufacturer’s instructions, with 10 ng cDNA as input. The primer sequences were as follows: *mir-103*, forward 5′-agcagcattgtacagggctatga-3′, reverse 5′-gaatcgagcaccagttacg-3′; *Pdk4*, forward 5′-gcatttctactcggatgctcatg-3′, reverse 5′-ccaatgtggcttgggtttcc-3′; *Ldha*, forward 5′-ggaaggaggttcacaagcag-3′, reverse 5′-acccgcctaaggttcat-3′; *Ndufb3*, forward 5′-acagacagtggaaaattgaaggg-3′, reverse 5′-gcccatgtatctccaagcct-3′; *Cyc1*, forward 5′-gcattcggaggggtttccag-3′, reverse 5′-ccgcatgaacatctcccca-3′; *Sdhb*, forward 5′-aatttgccatttaccgatggga-3′, reverse 5′-agcatccaacaccataggtcc-3′; and *Ucp3*, forward 5′-ggatttgtgccctcctttctg-3′, reverse 5′-cattaaggccctcttcagttgct-3′. miR-103/107 qPCR data are shown as miR-103 alone since the primer set cannot distinguish between the two miRNA paralogs.

### Western Blotting

Protein lysates were prepared from snap-frozen tissues in 500 μL radio-immunoprecipitation assay (RIPA) SDS (50 mM Tris-HCl, 150 mM sodium chloride, 0.1% SDS, 0.5% sodium deoxycholate, 1% NP-40, proteinase inhibitor cocktail (Roche), and 0.5 mM sodium orthovanadate) in the TissueLyser LT (QIAGEN, Venlo, the Netherlands). Cell lysates from nRCMs were prepared in 2× sample buffer (62.5 mM Tris-HCl, 50 mM dithiothreitol, 10% glycerol, 2% SDS, and 0.01% bromophenol blue). Total protein content of cell and tissue lysates was determined using the micro bicinchoninic acid (microBCA) protein assay kit (Thermo Fisher Scientific, Waltham, MA). 20 μg protein from tissue or cell lysate was resolved on SDS-PAGE polyacrylamide gel. The PageRuler protein ladder (Thermo Fisher Scientific, Waltham, MA) was used as a molecular weight reference. Electrophoresis was performed in a Mini-PROTEAN setup (Bio-Rad, Hercules, CA) for 1.5 h at 110 V. Proteins were transferred to a 0.45-μM polyvinylidene fluoride (PVDF) membrane (Merck Millipore, Billerica, MA) at 110 V/200 mA for 1 h. The membrane was blocked in 5% milk or BSA in Tris-buffered saline with 0.1% Tween for 1 h at room temperature, and it was incubated with primary antibody overnight at 4°C with gentle shaking.

The antibodies against Phospho-Troponin I (Ser23/24, 4004, Cell Signaling Technology, Danvers, MA, USA), total Troponin I (4002, Cell Signaling Technology, Danvers, MA, USA), phospho-phospholamban (Ser16/Thr17, 8496), and total phospholamban were purchased from Cell Signaling Technology (14562, Danvers, MA, USA); the antibody against GAPDH was purchased from Millipore (CB1001, Burlington, VT, USA); and the antibody against Phospho-CAMK II (Thr 177) was purchased from Santa Cruz Biotechnology (28438, Heidelberg, Germany). Horseradish peroxidase (HRP)-linked secondary antibodies anti-mouse immunoglobulin G (IgG), HRP-linked (7076, Cell Signaling Technology, Danvers, MA, USA) and anti-rabbit IgG, HRP-linked (7074, Cell Signaling Technology, Danvers, MA, USA) were applied in TBS-T for 1 h at room temperature. Immunoreactivity was visualized by a homemade enhanced chemiluminescence reagent (enhancer: 11 mg P-coumaric Acid and 10 mL DMSO; substrate: 10 mL 1 M Tris-HCl [pH 8.6], 25 mg sodium luminol, 21 μL 30% H_2_0_2_, and 90 mL distilled water), and immunoblot intensities were analyzed by densitometry with the software Image Studio Lite (Westburg, Leusden, the Netherlands).

### Histology

Paraffin-embedded 4-μm left ventricular sections were stained with H&E and picrosirius red, as described before,^33^ or GS lectin Biotinylated Griffonia Simplicifolia Lectin I (Burlingame, CA). Morphometric analyses were performed using a microscope (Leica DM2000; Leica, Wetzlar, Germany), camera (Leica DFC295 3Mpix CMOS color), and LAS Image Analysis software (Leica). Quantifications of fibrosis via via picrosirius red staining and of cardiomyocyte area via laminin staining were performed as previously described.^8^ Capillary density was analyzed in 8 lectin-stained sections/mouse at 40× magnification. Sections were analyzed with a capillary density assessment macro in ImageJ, from which tissue area (mm^2^), number of vessels, and number of vessels/mm^2^ were calculated.

### Transmission Electron Microscopy

Cardiac tissue was fixed with 3% glutaraldehyde in phosphate buffer and stored at 4°C. Tissue was washed with phosphate buffer and fixed with 1% osmium tetroxide in phosphate buffer containing 0.8% potassium ferricyanide at 4°C. Samples were dehydrated in acetone, infiltrated with Epon resin for 48 h, embedded, and polymerized at 60°C for 48 h. Ultrathin sections were obtained using a Leica Ultracut UC6 ultramicrotome (Leica Microsystems, Vienna, Austria) and mounted on Formvar-coated copper grids. Staining was performed with 2% uranyl acetate in water and lead citrate. The sections were observed in a Tecnai G2 Spirit transmission electron microscope equipped with a CCD Eagle 4kx4k camera (FEI, Eindhoven, the Netherlands). Five tissue samples per experimental group were selected and three pictures per sample were analyzed. Mitochondrial volume density was calculated as the mitochondria-containing fraction of a 32-by-32 grid placed on pictures of 1,900× magnification. To estimate the number and size distribution of all mitochondria within the cardiac tissue, mitochondria were counted, and the longest mitochondrial diameter was measured with ImageJ ROI-Manager on three pictures per sample at 1,900× magnification. The number of mitochondria in each size range was calculated as percentage of total mitochondria number for identical amounts of area in each sample. Mitochondrial ultrastructure was assessed at 6,800× magnification. Five tissue samples per experimental group were selected and three pictures per sample were analyzed.

### *In Vitro* Experiments

HL-1 cells were cultured according to Dr. Claycomb (Louisiana State University, New Orleans, LA).^34^ nRCMs were isolated and cultured in 6-well and 8-well microscopy μ-slides (80826, ibidi, Martinsried, Germany), as previously described.^35^ HL-1 cells or nRCMs were transfected with 30 nM miR-103/107 inhibitors (miRCURY LNA miRNA inhibitor, Exiqon; sequence 5′-CCCTGTACAATGCTGC-3′) or respective controls in combination with transfection reagent Lipofectamine 2000 (Thermo Fisher Scientific, Waltham, MA), according to the manufacturer’s instructions.

### HL-1 Substrate Uptake Experiments

Substrate uptake experiments were performed as previously described.^36^ Briefly, palmitate (coupled to BSA in a palmitate:BSA ratio of 1:3) and deoxy-D-glucose were added to final concentrations of 20 and 4 μM, respectively, with tracer amounts of [^14^C] palmitate and 2-deoxy-D-[^3^H]glucose. After 10 min, uptake was terminated and unbound substrate was removed by washing the cells with ice-cold depletion medium containing 0.2 mM phloretin. Cells were lysed by the addition of 1 M NaOH. Subsequently, incorporated glucose and palmitate were measured by scintillation counting of [^14^C] and [^3^H].

### Liquid Chromatography-MS

Tissue disruption and lysis were performed by use of Tissue Lyser II (QIAGEN) in a buffer containing 5 M Urea (GE Healthcare) in 50 mM Ammonium bicarbonate (ABC) (Sigma-Aldrich). The lysate was then reduced with 20 mM DTT for 45 min and alkylated with 40 mM iodoacetamide (IAM) for 45 min in the dark. The alkylation was terminated by 20 mM DDT to consume any excess IAM. Digestion was performed with a mixture of LysC and Trypsin (Promega), which was added at a ratio of 1:25 (enzyme to protein). After 2 h of digestion at 37°C in a water bath, the lysate was diluted with 50 mM ABC to 1 M Urea and further digested at 37°C overnight. The digestion was terminated by the addition of formic acid (FA) to a total of 1%. Biognosys (Biognosys, Schlieren, Switzerland) indexted Retention Time standards (iRTs) were added to each peptide sample according to the manufacturer’s instructions (required for the data-independent acquisition [DIA] analysis using Spectronaut). Peptide separation was performed on a Thermo Scientific (Dionex) Ultimate 3000 Rapid Separation UHPLC system equipped with an Acclaim PepMap C18 analytical column (2 μM, 100Å, 75 μM × 150 mm). Peptide samples were first desalted on an online installed C18 trapping column. After desalting, peptides were separated on the analytical column with a 90-min linear gradient from 5% to 35% Acetonitrile (Biosolve) with 0.1% FA at 300 nL/min flow rate. The UHPLC system was coupled to a Q Exactive HF mass spectrometer (Thermo Scientific).

Data-dependent acquisition (DDA) settings were as follows: full MS scan between 375 and 1,500 m/z at a resolution of 120,000 followed by tandem MS (MS/MS) scans of the top 15 most intense ions at a resolution of 30,000. The HRM DIA method consisted of a survey full MS scan at 120,000 resolution at 350–1,650 m/z. Then 58 DIA windows were acquired at 30,000 resolution. For protein identification, the DDA spectra were analyzed with Proteome Discoverer (PD) version 2.1.1.21. Within the PD software, the search engine Sequest was used with the SwissProt mouse database (*Mus musculus*; SwissProt TaxID = 10090; version (v.)2017-01-18) and the Biognosys iRT peptide sequences. The database search was performed with the following settings: enzyme was trypsin, a maximum of 2 missed cleavages, minimum peptide length of 6, precursor mass tolerance of 10 ppm, fragment mass tolerance of 0.02 Da, dynamic modifications of methionine oxidation and protein N terminus acetylation, and static modification of cysteine carbamidomethylation. The DDA measurements were used to create a spectral library using spectral library generation in Spectronaut 9 (Biognosys, Schlieren, Switzerland). Only identifications with false discovery rates (FDR) of maximum 1% at peptide and protein levels were taken into account for spectral library generation. For protein quantitation, the DIA data were analyzed with Spectronaut 9, with the manufacturer’s recommended default settings. The DDA data were analyzed with Proteome Discoverer 2.2 (Thermo Scientific).

### Metabolic Flux Analysis

Mitochondrial stress test and glycolytic stress test were performed on 4-day cultured primary nRCMs by real-time extracellular flux analyses using a Seahorse XF96 flux analyzer (Seahorse Bioscience, North Billerica, MA). OCR and ECAR were measured as previously described.^37^ For the mitochondrial stress OCR, an indicator of mitochondrial respiration was measured.^37^ Cells were seeded at a density of 50,000 cells/well in an XF96 cell culture microplate (Agilent). On the day of the measurement, cells were washed with XF Mitochondrial assay medium and placed in a 37°C incubator without CO_2_ for 1 h. An automated Seahorse XF96 protocol consisted of 20-min calibration and equilibration, followed by synchronized injection of drugs and/or reagents at optimized concentrations in each of 3 ports (mixing 4 min followed by a measurement of 4 min). Injection ports were loaded with the following: Oligomycin (ATP synthase inhibitor) at 1 μM, FCCP (oxidative phosphorylation uncoupler) at 0.5 μM, and a mixture of 1 μM Rotenone (respiratory complex I inhibitor) and 1 μM Antimycin A (respiratory complex III inhibitor), as described previously.^37^ OCR was recorded and averaged three times for each conditional cycle. For the glycolysis stress test, ECAR, a surrogate for anaerobic glycolysis, was measured using the Seahorse XF96 flux analyzer. Cells were seeded at a density of 70,000 cells/well in a XF96 cell culture microplate (Agilent). On the day of the measurement, cells were washed with XF Glycolytic assay medium and placed in a 37°C incubator without CO_2_ for 1 h prior to the assay. Injection ports were loaded with the following: glucose (10 mM), Oligomycin (2 μM) to stimulate anaerobic glycolysis for ATP production, and 2-deoxyglucose (2-DG) (100 mM) to inhibit glycolysis as described previously.^37^ After assay completion, total protein content of cell lysates was determined using the microBCA protein assay kit (Thermo Fisher Scientific, Waltham, MA).

### Mitochondrial Live-Cell Imaging

Live imaging of mitochondria was performed on primary nRCMs cultured and transfected in gelatin-coated 8-well microscopy μ-slides (80826, ibidi, Martinsried, Germany). Prior to imaging, cells were incubated with 250 nM TMRM (T668, Thermo Fisher Scientific, Waltham, MA), 3 μg/mL Calcein AM (C1430, Thermo Fisher Scientific, Waltham, MA), and 2.5 μg/mL Hoechst (H21486, Thermo Fisher Scientific, Waltham, MA) in phenol red free medium for 30 min at 37°C and 5% CO_2_ to stain mitochondria, cytoplasm, and nuclei, respectively. TMRM is utilized to monitor mitochondrial membrane potential and mitochondrial network structure. In healthy mitochondria, ETC and ATP synthesis are efficiently coupled, however, defects in the OXPHOS system affect mitochondrial membrane potential. If membrane potential is compromised, the retention of TMRM within mitochondria is hindered and the detectable fluorescent signal decreases. Cells were washed 3 times with PBS and incubated in phenol red free medium.

Mitochondria were observed with a CorrSight fluorescence microscope using a Zeiss 63× numerical aperture (NA) 1.4 objective, an Andromeda spinning-disc module, and a Hamamatsu ORCA-Flash4.0 V2 camera, resulting in a pixel size of 103 nm (FEI, Eindhoven, the Netherlands), in a chamber at 37°C and 5% CO_2_. For each experimental condition, 10 fields of view were imaged. Excitation lasers, with wavelengths of 405, 488, and 561 nm, and emission filters, with wavelengths of 446, 523, and 600 nm, were used for visualizing the nuclei, cytosol, and mitochondria, respectively. The z stacks that contained 100 images each were placed at Nyquist Z-distance (170 nm) to image whole cardiomyocytes. The z stacks underwent deconvolution using the Constrained Inference for Linear Mixed Effects Models (CLME) algorithm of Huygens Professional (SVI Hilversum). Cells were segmented manually on top of maximum-intensity projections of the channel-merged z stacks for the analysis of 3–6 cells/field of view. Image quantification was performed as described before,^38^ with the exception that our analysis was performed in 3 dimensions and was written in MATLAB 2017 (MathWorks) complemented with the DipImage toolbox (TU Delft). In short, all channels were first filtered both with a top-hat and a 3D median filter, masked using thresholding at a constant value, and segmented automatically. The volume of the cytoplasm was calculated by extracting the hole-filled nuclear volume (as defined by the Hoechst staining) from the hole-filled cytosolic volume, which reflects the cell volume (as defined by the Calcein staining).

### Statistical Analyses

Data are presented as mean ± SEM unless specified otherwise. Comparisons between 2 groups were performed with the 2-tailed Student’s t tests. For comparisons of more than 2 groups, 1-way ANOVA was used, followed by post hoc testing with Bonferroni correction for more groups. For comparisons between the same groups over time, repeated-measures ANOVA was used, followed by post hoc testing with Bonferroni correction. Values of p < 0.05 were considered statistically significant.

## Author Contributions

Conceptualization, B.S., M.v.B., and M.R.; Investigation, M.R., J. Lumens, J. Lecomte, P.C., R.V., A.R.K., R.v.L., W.V., Y.L., J.J.F.P.L., R.M., and B.C.-P.; Methodology, M.R., J. Lumens, J. Lecomte, P.C., R.V., A.R.K., R.v.L., W.V., B.C.-P., R.M., and K.K.; Software, J. Lumens, R.M., B.C.-P., and K.K.; Formal Analysis, B.S., M.v.B., and M.R.; Writing – Original Draft, M.R.; Writing – Review & Editing, B.S., M.v.B.,M.R., and A.R.K.; Funding Acquisition, B.S., M.v.B., and S.H.; Supervision, B.S. and M.v.B.

## Conflicts of Interest

There is no conflict of interest to disclose.

## Supplemental Information

Document S1. Figures S1 and S2 and Tables S1 and S2

Document S2. Article plus Supplemental Information
