# Supplementary material for: AntagomiR-103 and -107 Treatment Affects Cardiac Function and Metabolism
Source: Mol Ther Nucleic Acids. 2018 Dec 22;14:424–37. doi: 10.1016/j.omtn.2018.12.010 (PMC6365487; doi:10.1016/j.omtn.2018.12.010)
Supplement: Document S1. Figures S1 and S2 and Tables S1 and S2 [file mmc1.pdf]

OMTN, Volume *14*

Supplemental Information

AntagomiR-103 and -107 Treatment Affects Cardiac Function and Metabolism

Monika Rech, Annika R. Kuhn, Joost Lumens, Paolo Carai, Rick van Leeuwen, Wouter Verhesen, Robin Verjans, Julie Lecomte, Yilin Liu, Joost J.F.P. Luiken, Ronny Mohren, Berta Cillero-Pastor, Stephane Heymans, Kèvin Knoops, Marc van Bilsen, and Blanche Schroen

Supplemental information

Supplemental figures

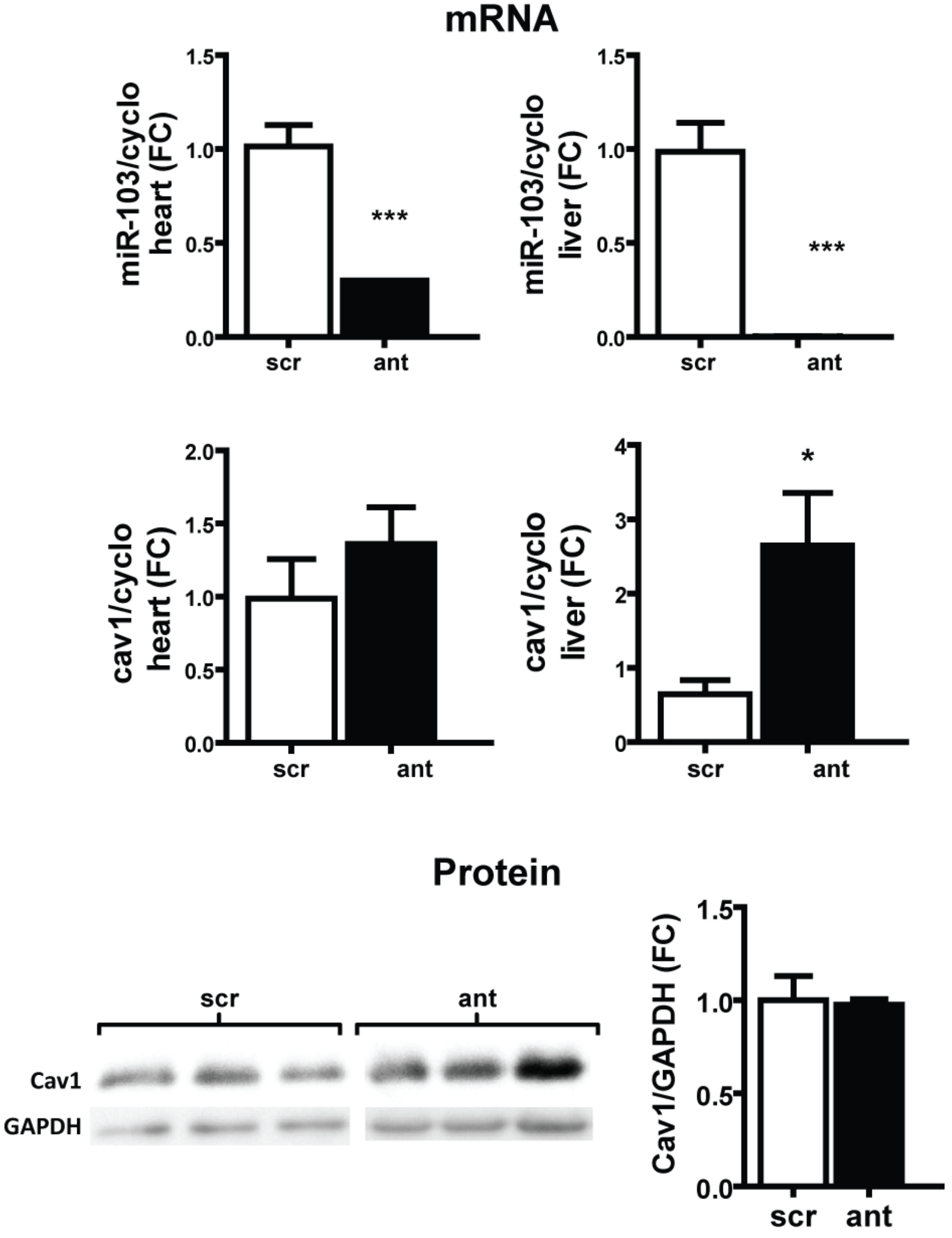

Figure S1. A) ***MiR-103*** **and** ***Cav1*** **gene expression in heart and liver of animals treated with scrambled control or antagomiR-103/107, * p<0.05 vs scrambled, *** p<0.001 vs scrambled, n=7 per group; B) representative western blot and quantification of Cav1 and GAPDH of the lysates of hearts treated with antagomiR-103/107 or scrambled, n=3 per group; data are shown as mean±SEM, scr, scrambled control, ant; antagomiR-103/107.**

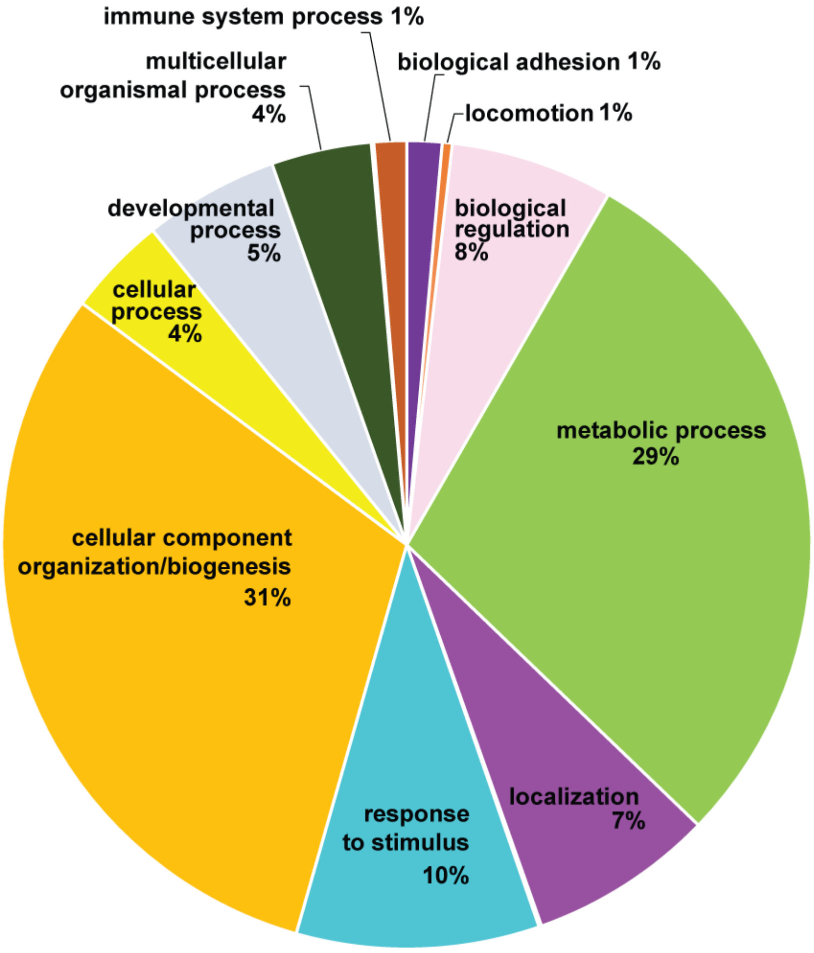

Figure S2. Panther GO-slim biological processes annotation chart for predicted targets of miR-103/107. Total predicted genes: 838; total number of process hits: 1397

Supplemental tables

Table S1. Hematological and immunological analysis in plasma of scrambled or antagomiR-103/107 treated mice at week 3.

Immune formula	Scrambled		AntagomiR	
	Mean	± SEM	Mean	± SEM
**WBC (10^3/µL)**	**5.17**	**0.24**	**3.94***	**0.33**
**Neutrophils (10^3/µL)**	0.33	0.03	0.27	0.04
**% Neutrophils**	6.54	0.76	6.85	0.42
**Lymphocytes (10^3/µL)**	**4.64**	**0.27**	**3.48***	**0.28**
**% Lymphocytes**	89.54	1.61	88.30	0.91
**Monocytes (10^3/µL)**	0.04	0.01	0.04	0.01
**% Monocytes**	0.78	0.31	0.90	0.19
**Eosinophils (10^3/µL)**	0.01	0.00	0.03	0.03
**% Eosinophils**	0.22	0.04	0.87	0.64
**Basophils (10^3/µL)**	0.15	0.03	0.12	0.02
**% Basophils**	2.91	0.62	3.08	0.21
**RBC (M/uL)**	6.10	0.18	6.58	0.11
**HGB (g/dL)**	8.97	0.26	9.68	0.18
**HCT (%)**	48.29	1.48	52.23	0.85
**MCV (fL)**	79.11	0.47	79.38	0.35
**MCH (pg)**	14.71	0.10	14.70	0.07
**MCHC (g/dL)**	18.59	0.06	18.53	0.06
**RDW (%)**	15.77	0.51	17.07	0.59
**PLT (K/dL)**	853.29	20.48	823.17	32.20
**MPV (fL)**	5.84	0.16	5.82	0.07
**PCT (%)**	0.50	0.03	0.48	0.02
**PDW (GSD)**	16.94	0.23	16.93	0.21

n=7 per group; * $p<0.05$ vs scrambled

Table S2. Mass spectrometry analysis of heart apex of scrambled or antagomiR-103/107 treated mice.

Protein	UniProt ID	Unique Peptides	Log2 Ratio	Q-value	Ant/ Scr	Ant [-]	Scr [-]	Data Points
**26S proteasome non-ATPase regulatory subunit 7 [OS=Mus musculus]**	P26516	4	0.52	1.81E-02	1.43	18129.9	12684.1	4
**complement factor H [OS=Mus musculus]**	P06909	2	0.42	1.22E-02	1.34	5832.5	4345.8	2
**Hemopexin [OS=Mus musculus]**[a]	Q91X72	18	0.38	6.04E-11	1.30	248566.8	191392.4	23
**Peptidyl-prolyl cis-trans isomerase F, mitochondrial [OS=Mus musculus]**	Q99KR7	8	0.35	1.96E-02	1.27	24018.7	18844.0	9
**Complement C3 [OS=Mus musculus]**	P01027-1	11	0.28	1.10E-02	1.21	50356.1	41446.4	12
**Actin, alpha skeletal muscle [OS=Mus musculus]**	P68134	3	0.28	1.84E-02	1.21	167488.3	138069.6	6
**Cytochrome c, somatic [OS=Mus musculus]**	P62897	24	0.27	9.73E-17	1.20	2779866.3	2307895.8	41
**Acylphosphatase-2 [OS=Mus musculus]**	P56375	7	0.27	9.64E-04	1.20	122844.4	102199.0	8
**Aspartate aminotransferase, mitochondrial [OS=Mus musculus]**	P05202	41	0.24	5.26E-14	1.18	1101538.9	933398.8	62
**Fibrinogen gamma chain [OS=Mus musculus]**	Q8VCM7	10	0.24	3.18E-03	1.18	68054.0	57702.7	10
**Myosin-7 [OS=Mus musculus]**	Q91Z83	65	0.20	6.88E-05	1.15	1948494.3	1701126.8	96
**Fatty acid-binding protein, heart [OS=Mus musculus]**	P11404	23	0.18	3.80E-05	1.13	5532021.5	4883083.0	50
**Protein S100-A1 [OS=Mus musculus]**	P56565	5	0.18	1.71E-02	1.13	183583.0	162465.1	8
**Actin, alpha cardiac muscle 1 [OS=Mus musculus]** [a]	P68033	60	0.15	2.20E-03	1.11	61439824.0	55271280.0	163
**myosin regulatory light chain 2, ventricular/cardiac muscle isoform [OS=Mus musculus]**	P51667	37	0.15	9.10E-03	1.11	7096360.5	6411138.5	83
**Translationally-controlled tumor protein [OS=Mus musculus]**	P63028	10	0.14	1.10E-02	1.11	297646.4	269287.2	13
**Myosin-4 [OS=Mus musculus]**	Q5SX39	14	0.14	3.85E-03	1.10	3429579.3	3111626.8	20
**Pregnancy zone protein [OS=Mus musculus]**	Q61838	13	0.12	9.46E-03	1.09	101444.3	93338.5	14
**Myoglobin [OS=Mus musculus]** [a]	P04247	36	0.12	3.72E-03	1.08	14948670.0	13778867.0	88
**Acyl-coenzyme A thioesterase 13 [OS=Mus musculus]**	Q9CQR4	13	0.10	1.04E-02	1.07	1336000.1	1245060.6	23
**Telethonin [OS=Mus musculus]**	O70548	4	0.10	2.50E-03	1.07	153428.2	143335.0	4
**electron transfer flavoprotein subunit beta [OS=Mus musculus]**	Q9DCW4	35	0.06	2.78E-06	1.04	3497220.5	3361798.3	57
**Cytochrome b-c1 complex subunit 7 [OS=Mus musculus]**	Q9D855	18	0.05	2.73E-05	1.04	1626810.6	1571087.5	30
**ATP synthase subunit gamma, mitochondrial [OS=Mus musculus]**	Q91VR2	28	0.04	7.14E-05	1.03	1369158.4	1327708.0	44
**Laminin subunit alpha-2 [OS=Mus musculus]**	Q60675	60	0.03	7.67E-04	1.02	167368.0	164485.1	69
**glyceraldehyde-3-phosphate dehydrogenase [OS=Mus musculus]**	P16858	38	0.02	4.55E-04	1.01	6986970.0	6887551.5	79
**Beta-enolase [OS=Mus musculus]**	P21550	52	-0.01	3.02E-04	0.99	2640891.5	2665133.0	92
**Adenylate kinase isoenzyme 1 [OS=Mus musculus]**	Q9R0Y5-1	25	-0.02	2.39E-04	0.99	2043768.6	2069378.4	40
**Ornithine aminotransferase, mitochondrial [OS=Mus musculus]**	P29758	17	-0.03	1.64E-02	0.98	193450.4	197165.2	26
**Alpha-actinin-2 [OS=Mus musculus]**	Q9JI91	120	-0.03	8.73E-04	0.98	2255220.0	2309955.8	209
**Cysteine-rich protein 2 [OS=Mus musculus]**	Q9DCT8	16	-0.04	1.76E-02	0.97	630263.5	648074.9	27
**NADH dehydrogenase [ubiquinone] 1 alpha subcomplex subunit 2 [OS=Mus musculus]**	Q9CQ75	11	-0.05	1.33E-02	0.97	795473.6	821258.3	16
**Myosin-3 [OS=Mus musculus]**	P13541	9	-0.05	4.03E-03	0.97	1769777.5	1828452.1	11
**Cytochrome c oxidase subunit 6B1 [OS=Mus musculus]**	P56391	11	-0.05	5.96E-04	0.97	6591877.0	6821341.0	18
**Malate dehydrogenase, cytoplasmic [OS=Mus musculus]**	P14152	37	-0.05	8.40E-03	0.97	4713308.0	4881656.0	59
**Alpha-crystallin B chain [OS=Mus musculus]**	P23927	23	-0.06	1.23E-02	0.96	1099699.0	1147986.0	47

**ATP synthase subunit d, mitochondrial [OS=Mus musculus]**	Q9DCX2	21	-0.07	3.51E-03	0.95	2873328.3	3010304.5	37
**Aspartate aminotransferase, cytoplasmic [OS=Mus musculus]**	P05201	35	-0.07	1.52E-03	0.95	1689716.6	1777122.5	55
**Isocitrate dehydrogenase [NAD] subunit alpha, mitochondrial [OS=Mus musculus]**	Q9D6R2	34	-0.08	6.38E-04	0.95	1214956.3	1279973.0	57
**Malate dehydrogenase, mitochondrial [OS=Mus [a] musculus]**	P08249	39	-0.08	3.74E-09	0.95	15937558.0	16827072.0	74
**Cytochrome b-c1 complex subunit 2, mitochondrial [OS=Mus musculus]**	Q9DB77	34	-0.08	7.17E-03	0.94	2317813.8	2453957.8	58
**EH domain-containing protein 1 [OS=Mus musculus] [a]**	Q9WVK4	15	-0.08	3.45E-03	0.94	72112.0	76449.4	20
**Electron transfer flavoprotein subunit alpha, mitochondrial [OS=Mus musculus]**	Q99LC5	32	-0.09	1.52E-04	0.94	4521429.5	4796793.5	58
**NADH dehydrogenase [ubiquinone] flavoprotein 1, mitochondrial [OS=Mus musculus]**	Q91YT0	37	-0.09	3.95E-03	0.94	1150154.4	1222285.6	67
**Troponin I, cardiac muscle [OS=Mus musculus]**	P48787	31	-0.10	3.99E-04	0.93	5298699.0	5685937.0	49
**Cytochrome c oxidase subunit 2 [OS=Mus musculus]**	P00405	8	-0.10	1.64E-02	0.93	4445445.5	4772605.0	16
**Heterogeneous nuclear ribonucleoproteins C1/C2 [OS=Mus musculus]**	Q9Z204;-2;-3;-4;-5	5	-0.11	1.99E-02	0.93	39069.5	42063.2	5
**Carnitine O-palmitoyltransferase 2, mitochondrial [OS=Mus musculus]**	P52825	43	-0.11	1.84E-03	0.93	349260.2	376413.4	54
**Very long-chain specific acyl-CoA dehydrogenase, mitochondrial [OS=Mus musculus]**	P50544	48	-0.11	8.97E-03	0.93	1795736.5	1940154.9	69
**Protein FAM136A [OS=Mus musculus] [a]**	Q9CR98	5	-0.11	4.98E-03	0.92	46795.8	50645.9	7
**O-acetyl-ADP-ribose deacetylase MACROD1 [OS=Mus musculus]**	Q922B1	20	-0.12	9.49E-06	0.92	228717.3	247875.4	28
**EH domain-containing protein 4 [OS=Mus musculus] [a]**	Q9EQP2	14	-0.13	8.49E-06	0.92	179074.2	195609.8	15
**Dystroglycan [OS=Mus musculus]**	Q62165	5	-0.14	1.51E-03	0.91	17315.3	19054.6	6
**Peroxiredoxin-5, mitochondrial [OS=Mus musculus]**	P99029;-2	15	-0.14	3.71E-03	0.91	2688488.0	2962060.0	26
**Mitochondrial import inner membrane translocase subunit TIM44 [OS=Mus musculus]**	O35857	10	-0.14	2.32E-03	0.91	36331.5	40140.4	11
**Methylcrotonoyl-CoA carboxylase beta chain, mitochondrial [OS=Mus musculus]**	Q3ULD5	25	-0.15	1.07E-03	0.90	228112.0	252481.9	30
**Guanine nucleotide-binding protein G(I)/G(S)/G(T) subunit beta-2 [OS=Mus musculus]**	P62880	4	-0.15	1.83E-02	0.90	40107.8	44575.5	5
**Heterogeneous nuclear ribonucleoprotein U [OS=Mus musculus]**	Q8VEK3	7	-0.16	2.96E-03	0.90	24624.8	27453.8	11
**medium-chain specific acyl-CoA dehydrogenase, mitochondrial [OS=Mus musculus]**	P45952	38	-0.16	4.07E-03	0.90	3404054.5	3796175.8	65
**haloacid dehalogenase-like hydrolase domain-containing protein 2 [OS=Mus musculus]**	Q3UGR5-1	4	-0.16	1.18E-02	0.90	49349.2	55088.4	4
**Long-chain-fatty-acid--CoA ligase 1 [OS=Mus musculus] [a]**	P41216	55	-0.16	2.96E-08	0.89	849570.8	950392.8	73
**Hemoglobin subunit beta-2 [OS=Mus musculus]**	P02089	6	-0.16	1.58E-04	0.89	16437964.0	18395956.0	13
**Long-chain specific acyl-CoA dehydrogenase, mitochondrial [OS=Mus musculus] [a]**	P51174	36	-0.16	1.65E-02	0.89	4784476.5	5356746.5	58
**Heterogeneous nuclear ribonucleoprotein M [OS=Mus musculus]**	Q9D0E1;-2	8	-0.16	1.38E-03	0.89	41784.1	46842.8	8
**Bifunctional glutamate/proline--tRNA ligase [OS=Mus musculus]**	Q8CGC7	14	-0.17	5.65E-03	0.89	61504.2	69178.1	14
**Branched-chain-amino-acid aminotransferase, mitochondrial [OS=Mus musculus] [a]**	O35855	15	-0.19	4.74E-05	0.88	217851.4	248441.3	19
**Delta(3,5)-Delta(2,4)-dienoyl-CoA isomerase, mitochondrial [OS=Mus musculus]**	O35459	29	-0.19	5.16E-03	0.87	727510.5	831638.0	44
**Dihydrolipoyllysine-residue succinyltransferase comp of 2-oxoglutarate dehydrogenase complex, [OS=Mus musculus]**	Q9D2G2-1	28	-0.19	3.66E-03	0.87	2201266.3	2518189.5	38
**T-complex protein 1 subunit delta [OS=Mus musculus]**	P80315	4	-0.20	4.20E-03	0.87	13949.2	16061.8	4
**NADH dehydrogenase [ubiquinone] iron-sulfur protein 7, mitochondrial [OS=Mus musculus]**	Q9DC70	13	-0.22	6.39E-03	0.86	353860.3	411094.7	17
**40S ribosomal protein S11 [OS=Mus musculus]**	P62281	2	-0.22	7.82E-04	0.86	13002.6	15128.0	3
**NADH dehydrogenase [ubiquinone] 1 beta subcomplex subunit 10 [OS=Mus musculus] [a]**	Q9DCS9	21	-0.22	3.54E-03	0.86	734613.3	856187.3	32
**sorting and assembly machinery component 50 homolog [OS=Mus musculus]**	Q8BGH2	10	-0.22	1.68E-02	0.86	96777.8	112939.0	11
**Acyl-coenzyme A thioesterase 2, mitochondrial [OS=Mus musculus] [a]**	Q9QYR9	26	-0.23	1.49E-03	0.85	567183.2	663567.3	35

**Laminin subunit beta-1 [OS=Mus musculus]**	P02469	14	-0.23	2.37E-03	0.85	171563.8	200856.8	16
**fructosamine-3-kinase [OS=Mus musculus]**	Q9ER35	2	-0.23	4.66E-03	0.85	26484.9	31151.6	2
**EH domain-containing protein 2 [OS=Mus musculus]**	Q8BH64	18	-0.23	1.02E-03	0.85	133321.6	156871.3	25
**60S ribosomal protein L10-like [OS=Mus musculus]**	P86048;Q6ZWV3	5	-0.27	1.63E-05	0.83	65484.8	78754.8	5
**adiponectin [OS=Mus musculus]**	Q60994	3	-0.28	7.19E-03	0.82	22722.7	27560.4	3
**Lon protease homolog, mitochondrial [OS=Mus musculus]**	Q8CGK3	19	-0.28	2.78E-03	0.82	175859.7	213585.7	23
**40S ribosomal protein S2 [OS=Mus musculus]**	P25444	4	-0.29	3.19E-03	0.82	34421.7	41953.7	4
**Selenium-binding protein 1 [OS=Mus musculus]**	P17563	9	-0.29	5.88E-06	0.82	72690.8	88882.3	10
**NAD kinase 2, mitochondrial [OS=Mus musculus];**	Q8C5H8-1;-2;-3;-4	2	-0.30	7.84E-03	0.81	4520.9	5569.2	2
**Transketolase [OS=Mus musculus]**	P40142	9	-0.31	6.82E-03	0.81	20513.9	25462.9	9
**C-1-tetrahydrofolate synthase, cytoplasmic [OS=Mus musculus]**	Q922D8	3	-0.36	1.64E-02	0.78	5013.7	6419.4	3
**Heat shock protein HSP 90-alpha [OS=Mus musculus]**	P07901	8	-0.36	1.77E-02	0.78	180191.6	231620.6	11
**histone H3.2 [OS=Mus musculus]**	P84228	1	-0.36	8.62E-05	0.78	88267.4	113576.8	3
**40S ribosomal protein S8 [OS=Mus musculus]**	P62242	5	-0.38	1.73E-07	0.77	25016.8	32583.1	6
**60S ribosomal protein L7a [OS=Mus musculus]**	P12970	8	-0.41	1.73E-02	0.76	16516.1	21871.5	8
**60S ribosomal protein L32 [OS=Mus musculus]** [a]	P62911	4	-0.42	1.97E-02	0.75	8644.8	11577.0	5
**Catenin beta-1 [OS=Mus musculus]**	Q02248	5	-0.45	3.71E-03	0.73	9466.7	12930.9	5
**Histone H1.0 [OS=Mus musculus]**	P10922	5	-0.47	1.44E-05	0.72	23809.3	33064.0	5
**60S ribosomal protein L27a [OS=Mus musculus]**	P14115	5	-0.48	1.50E-02	0.72	37959.2	52889.9	5
**Aspartyl aminopeptidase [OS=Mus musculus]**	Q9Z2W0	3	-0.48	9.84E-04	0.72	28783.8	40217.2	3
**Histone H1.3 [OS=Mus musculus]**	P43277	11	-0.55	8.45E-05	0.68	74064.0	108330.6	16
**60S ribosomal protein L7 [OS=Mus musculus]**	P14148	6	-0.56	1.81E-02	0.68	9013.1	13247.8	6
**histone H4 [OS=Mus musculus]** [a]	P62806	11	-0.57	1.65E-09	0.67	1654453.1	2456581.3	16
**Alpha-actinin-3 [OS=Mus musculus]**	O88990	2	-0.61	1.82E-03	0.66	18781.9	28585.9	3
**60S ribosomal protein L14 [OS=Mus musculus]**	Q9CR57	3	-0.65	1.88E-03	0.64	10302.6	16135.4	4
**40S ribosomal protein S25 [OS=Mus musculus]**	P62852	3	-0.69	1.90E-03	0.62	37913.7	60990.8	3
**Histone H3.1 [OS=Mus musculus]**	P68433	14	-0.69	1.02E-19	0.62	1074207.0	1730754.3	18
**Histone H3.3C [OS=Mus musculus];histone H3.3 [OS=Mus musculus]**	P02301;P84244	2	-0.72	1.21E-03	0.61	39108.6	64587.5	3
**Glutathione reductase, mitochondrial [OS=Mus musculus]** [a]	P47791;P47791-2	2	-0.81	6.20E-03	0.57	8223.1	14383.9	2
**Myosin-1 [OS=Mus musculus]**	Q5SX40	1	-0.83	3.74E-06	0.56	262413.5	465269.9	2
**Eukaryotic translation initiation factor 3 subunit I [OS=Mus musculus]**	Q9QZD9	3	-0.94	1.96E-02	0.52	3608.7	6943.2	4

n=3 per group analyzed in technical triplicates. Ant [-], average of antagomiR-103/107; Scr [-], average of scrambled control; [-], no unit; Log2 ratio represents the average of the Log2 ratios of the 3 samples per group; [a], target of miR-103/107 based on TargetScan prediction.
